# Determinants of insecticide-treated net ownership and utilization among pregnant women in Nigeria

**DOI:** 10.1186/1471-2458-12-105

**Published:** 2012-02-06

**Authors:** Augustine Ankomah, Samson B Adebayo, Ekundayo D Arogundade, Jennifer Anyanti, Ernest Nwokolo, Olaronke Ladipo, Martin M Meremikwu

**Affiliations:** 1 Society for Family Health, 8 Port Harcourt Crescent, Area 11 Abuja, Nigeria; 2 College of Medical Sciences, University of Calabar, Calabar, Nigeria

## Abstract

**Background:**

Malaria during pregnancy is a major public health problem in Nigeria leading to increase in the risk of maternal mortality, low birth weight and infant mortality. This paper is aimed at highlighting key predictors of the ownership of insecticide treated nets (ITNs) and its use among pregnant women in Nigeria.

**Methods:**

A total of 2348 pregnant women were selected by a multi-stage probability sampling technique. Structured interview schedule was used to elicit information on socio-demographic characteristics, ITN ownership, use, knowledge, behaviour and practices. Logistic regression was used to detect predictors of two indicators: ITN ownership, and ITN use in pregnancy among those who owned ITNs.

**Results:**

ITN ownership was low; only 28.8% owned ITNs. Key predictors of ITN ownership included women who knew that ITNs prevent malaria (OR = 3.85; *p *< 0001); and registration at antenatal clinics (OR = 1.34; *p *= 0.003). The use of ITNs was equally low with only 7.5% of all pregnant women, and 25.7% of all pregnant women who owned ITNs sleeping under a net. The predictors of ITN use in pregnancy among women who owned ITNs (N = 677) identified by logistic regression were: urban residence (OR = 1.87; *p *= 0.001); knowledge that ITNs prevent malaria (OR = 2.93; *p *< 0001) and not holding misconceptions about malaria prevention (OR = 1.56; *p *= 0.036). Educational level was not significantly related to any of the two outcome variables. Although registration at ANC is significantly associated with ownership of a bednet (perhaps through free ITN distribution) this does not translate to significant use of ITNs.

**Conclusions:**

ITN use lagged well behind ITN ownership. This seems to suggest that the current mass distribution of ITNs at antenatal facilities and community levels may not necessarily lead to use unless it is accompanied by behaviour change interventions that address the community level perceptions, misconceptions and positively position ITN as an effective prevention device to prevent malaria

## Background

Maternal mortality in Nigeria is among the highest in the world, with a mortality ratio exceeding 800 per 100,000 live births in most zones of the country [1]. Malaria directly accounts for about 11% of all maternal deaths, and indirectly contributes to additional 11% of maternal deaths mainly by being a leading cause of anaemia in pregnancy [2]. Malaria infection in pregnancy affects both the mother and her unborn child. Placental infections compromise foetal nutrition leading to intrauterine foetal growth retardation and low birth weight [3]. Placental infection and other deleterious effects of malaria are worse in first pregnancies than in subsequent ones [4]. Low birth weight caused by malaria in pregnancy is estimated to account for over 100,000 infant deaths in Africa annually [5]. This explains why national efforts to reduce the high maternal and infant mortality place high premium on effective control of malaria in pregnancy.

In line with the recommendation of the World Health Organization, the Nigerian national strategy for malaria control in pregnancy focuses on three approaches: providing prompt access to effective treatment; use of insecticide treated nets (ITNs); and intermittent preventive treatment (IPT) with sulphadoxine-pyrimethamine [6,7]. Consistent use of ITNs in pregnancy has been shown by several randomized controlled trials to produce beneficial maternal and infant outcomes [8].

Nigeria has promoted ITN use in pregnancy along with other evidence-based interventions for malaria control since the Abuja Malaria Summit [9] but levels of ITN utilization by pregnant women and other vulnerable population groups have remained low [10-12]. The results of the most recent Demographic and Health Survey conducted in the country showed utilization rates below 10% [13].

A recent synthesis of data from national malaria control programmes has also shown that levels of utilization of ITN by pregnant women in many other sub-Saharan African countries remain far below national and global strategic targets [14,15]. The use of ITNs in Nigeria is very low even by sub-Saharan African standards. The percentage of children under 5 in Nigeria who slept under ITNs increased only marginally from 3.3% in 2004 to 6% in 2008 [13,14]. There have been recent efforts to increase access to ITNs through mass distribution programmes but there are concerns that ITN utilization may still lag behind access as has been severally reported [16-18]. Several community level studies have identified poor perceptions about, and low use of ITNs in several African countries including Ghana [19], Tanzania [20] and Nigeria [21-24].

There is need to study factors capable of influencing consistent utilization of ITN especially by vulnerable population groups like under-five children and pregnant women in various settings. The reported studies on bed net utilization in Nigeria are mostly limited to caregivers of children below five years of age (12, 23), and pregnant women have often not been the focus of study. Nigerian studies that have specifically studied ITN use by pregnant women have been mostly facility-based and involved relatively small samples. A notable example is the survey conducted in Osogbo southwest Nigeria which reported low ITN use in a sample of 328 pregnant women but did not explore possible determinants (24).

The present paper reports the findings of multivariate analysis of data from a large-scale national, house-to-house survey of pregnant women aimed at identifying some determinants of ownership of bednets and the use of bednets among pregnant women who own bednets The core theoretical basis for the study is the health belief model (25). This theoretical model hypothesizes that personal health-related action is determined by the existence of sufficient motivation or health concern, perceived threat of serious outcome and the belief that recommended health action will reduce or eliminate the perceived threat (25, 26).

## Methods

### Study area and population

#### Study design

The study was a population-based household cross-sectional survey undertaken in October-December 2008. Multi-stage cluster sampling design was used to select pregnant women. The selection process was aimed at achieving a representative sample of study participants at the state level.

Structured questionnaire based on thematic areas was pilot-tested before use. The study questionnaires elicited information on demographic socio-economic, knowledge, attitude, belief, health care-seeking behaviour and the various issues related to prevention and treatment of malaria in pregnancy. Interviewers were selected based on their understanding of both English and the local languages of the communities where they worked. Interviewers were trained to understand and appropriately apply all aspects of the questionnaire and interview process. Approval was sought and received from each State Ministry of Health, community leaders, and informed consent was obtained from the heads of households and the respondents. All interviews took place in the homes of respondents and confidentiality was assured.

#### Sampling procedure

Out of the 36 states that make up Nigeria, 21 (including the Federal Capital Territory) were included in this survey. These 21 states were selected because they had fairly comparable access to resources for state-wide implementation of interventions for malaria control (including distribution of free ITNs to pregnant women). Eighteen of the states were the only states in the country supported by the Global Fund grants at the time of the survey while the other three were receiving comparable support from leading international development partners. In addition to the donor support, all the states received statutory funding from annual government budgets. The Malaria Control Programme in all these states followed a National Strategic Plan that derived largely from the Global Malaria Control Strategy and Roll Back Malaria. Resources provided for malaria control include long lasting insecticide treated bed nets, artemisinin-based combination treatments (ACTs) and intermittent preventive treatment in pregnancy.

We used a multi-stage sampling procedure to select 20 clusters per State. To achieve this, we used enumeration area (EA) maps for the Local Government Areas (LGAs) and communities selected from each state. The EA maps were obtained from the National Population Commission. A cumulative measure of the size was obtained using the appropriate power calculation formulae. We estimated that 20 clusters of approximately 18 households per cluster would yield a minimum of 120 pregnant women per state. Women of reproductive age within the selected households were identified and listed. From the household listing, women of reproductive age were identified and further screened to select those who were pregnant and these were subsequently interviewed. The total sample size was 2348 pregnant women.

### Data analysis

Data were entered using the Census Surveys Professional software (CSPro), and were exported to, managed and analyzed using SPSS 18.0^®^. We employed a logistic regression to examine the factors that predict two outcomes; first, bednet ownership among the total sample of pregnant women, and second, the use or non-use of ITNs among pregnant women who own bednets in Nigeria [25,26], This permitted us to compute the probability that a respondent used or did not use ITNs controlling for other covariates, including demographics and knowledge indicators. Conceptualization and construct of variables and analytical models took into consideration the key concepts of the "health belief theoretical model" namely perceived susceptibility, severity of perceived threat and perceived benefit of recommended health action which, in the context of this study refers to ownership and use of ITNs during pregnancy (25, 26).

#### Dependent variables

The study has two dependent variables: net ownership; and the use of bednets by pregnant women the night before the study. In this study, the definition of ITNs includes long lasting insecticide treated nets and all other insecticide treated nets. Therefore, all insecticide treated nets, whether long lasting insecticide treated nets or retreated nets are referred to in this paper as ITNs. Non treated nets were not included in the definition of use.

#### Independent variables

The independent variables in this paper are: geo-political zones (Nigeria has six geo-political divisions: North-East, North-Central, North-West, South-East, South-South and South-West), residence (urban or rural), age, education, knowledge about cause of malaria (i.e. knowing that it is transmitted by mosquitoes). Composite measure for 'misconceptions about causes of malaria', and 'misconceptions about prevention of malaria' were based on variables identified from previous studies [23,24]. These included the attribution of malaria to 'staying too long in the sun', 'not resting enough/no sleep', 'drinking too much alcohol/beer', 'witchcraft/juju', 'eating too much palm/groundnut oil', and 'excessive laborious work. Knowledge about malaria prevention was created as a composite indicator measuring how pregnant women responded to whether or not malaria could be prevented by taking any of the following actions: taking malaria medicine every day/weekly; Clearing bushes around the house, destroying places where mosquitoes breed (e.g. stagnant water), sleeping under insecticide treated net, using mosquito repellent creams/mosquito coils, keeping out of the sun, using insecticide to kills mosquitoes before sleeping in a room, using nets on windows and doors of the house, and using brooms/rackets. One item - knowledge that ITNs prevent against malaria- was analysed separately.

The analysis of the determinants of each of the two dependent variables was done separately. Both bivariate and multivariate analyses were undertaken to explore the determinants of bednet ownership and the statistical significance was based on a p-value of less than 0.05. At an exploratory data analysis stage, several models were explored. Test of goodness-of-fit was based on Hosmer and Lemeshow test (HL); models with p > 0.05 were assumed to be good and fit well to the data. Several models were explored but the model with the best fit according to HL was selected for reporting (p-value = 0.832).

## Results

### General characteristics of respondents

Table 1 presents the information about the background characteristics of the two groups of respondents: those own bednets (N = 677) and those who do not (N = 1,671). The majority of pregnant women who own or did not own bednets were resident in rural areas. Most of the pregnant women interviewed were either full time housewives or self-employed (71.6% among those who did not won bednets, and 68.6% for those who did). This was true whether they own or did not own bednets.

**Table 1:** General characteristics of survey respondents according to ownership of a bednet

Characteristics	Respondents who do not own a bednet				Respondents who own a bednet			
	
	Rural	Urban	Total (%)	Total Number	Rural	Urban	Total (%)	Total Number
**Age**								
Below 26 years	48.1	41.4	45.0	752	46.7	36.8	42.4	287
26 years and above	51.9	58.6	55.0	919	53.3	63.2	57.6	390
**Education**								
None	27.2	12.4	20.4	341	18.4	14.2	16.5	112
Qur'anic only	15.9	10.0	13.2	221	16.8	12.5	14.9	101
Primary	24.1	20.7	22.6	377	28.3	17.2	23.5	159
Secondary	28.5	44.7	35.9	600	29.4	41.6	34.7	235
Higher	4.2	12.2	7.9	132	7.1	14.5	10.3	70
**Geopolitical Zones**								
North West	19.5	16.0	17.9	299	22.0	25.0	23.3	158
North East	20.9	15.4	18.4	307	20.7	22.3	21.4	145
North Central	22.0	18.2	20.3	339	16.8	16.6	16.7	113
South West	8.9	25.3	16.4	274	5.5	17.9	10.9	74
South East	13.4	10.2	11.9	199	17.6	10.8	14.6	99
South South	15.3	15.0	15.1	253	17.3	7.4	13.0	88
**Occupation**								
Formal job	2.3	4.6	3.4	56	5.0	9.8	7.1	48
Self employed	32.7	35.5	34.0	568	24.1	31.4	27.3	185
Housewives	37.8	37.4	37.6	628	36.7	37.2	36.9	250
Others	27.2	22.5	25.1	419	34.1	21.6	28.7	194
**ANC Attendance**								
Registered	45.6	56.8	50.7	848	57.2	61.5	59.1	400
Did not register	54.4	43.2	49.3	823	42.8	38.5	40.9	277

**Total**	**54.0**	**46.0**	**100.0**	**1671**	**56.3**	**43.7**	**100.0**	**677**

A majority were literate. The proportion who had secondary education was fairly high (28.5% rural, 44.7% urban) among pregnant women who did not own bednets and 29.4% in rural and 41.6% urban among respondents who own bednet. Only about a fifth of those who did not own bednets had no formal education, compared with 16.5% of those who did. Since some pregnant women obtain their ITNs from visits to antenatal healthcare facilities, the study obtained information on antenatal visits. Of the 1671 pregnant women who did not own bednets, slightly over one-half (50.7%), had registered for antenatal care but a higher proportion (59.1%) among those who own bednets had done so.

### Bivariate analysis of ownership of ITNs and independent variables

Of the total number of 2,348 pregnant women interviewed, as shown in Table 2, only 28.8% (677 women) reported to own bednets. At the first stage of bivariate analysis, considering the fact that bednet ownership of bednets is often a precursor to use, we analysed the complete dataset comprising of 2,348 pregnant women to identify factors associated with bednet ownership. As shown in Table 2, bednet ownership was found to be significantly associated with geopolitical zones (*p *< .0001). Thus while net ownership among pregnant women stood at 34.6% in North West, the figure for the South West was 21.3% (i.e. lowest among all the geopolitical zones). Pregnant women who had registered for antenatal care were also more likely to own bednets (32.1% vs. 25.2%; *p *< 0.0001). Pregnant women who perceived malaria in pregnancy to be harmful were more likely to own bednets (*p *= 0.037). Pregnant women who had the correct knowledge of how to prevent malaria (an index variable) were more likely to own bednets (*p *< 0.0001). Similarly, women who knew that ITNs prevent malaria were more likely to own ITNs (49.6% vs. 21.9%; *p *< .0001). Some key background characteristics: residence (rural-urban), education, and age were found not to be significantly related to bednet ownership. Also there was no significant difference in net ownership between women who held misconceptions and those who did not (*p *= 0.408).

**Table 2:** Bivariate analysis of bednet ownership and use (among those who own) among pregnant women with independent variables

Characteristics	% of ownership of ITN (N_1 _= 2348)			% of ITN use in pregnancy (N_2 _= 677)		
	
	Yes	No	P-value	Yes	No	P-value
**Locality**						
Rural	29.7	70.3	0.337	20.7	79.3	0.001
Urban	27.8	72.2		32.1	67.9	
**Age of caregivers**						
Below 26 years	27.6	72.4	0.252	28.6	71.4	0.143
26 years and above	29.8	70.2		23.6	76.4	
**Education**						
None	24.7	75.3	0.077	23.2	76.8	0.317
Qur'anic only	31.4	68.6		21.8	78.2	
Primary	29.7	70.3		29.6	70.4	
Secondary	28.1	71.9		23.8	76.2	
Higher	34.7	65.3		32.9	67.1	
**Geopolitical Zones**						
North West	34.6	65.4	<0.0001	30.4	69.6	0.170
North East	32.1	67.9		20.7	79.3	
North Central	25.0	75.0		29.2	70.8	
South West	21.3	78.7		28.4	71.6	
South East	33.2	66.8		18.2	81.8	
South South	25.8	74.2		27.3	72.7	
**Registered for ANC**						
No	25.2	74.8	<0.0001	22.7	77.3	0.143
Yes	32.1	67.9		27.8	72.3	
**Risk of malaria in pregnancy**						
Perceived it is not harmful	23.3	76.7	0.037	20.3	79.7	0.009
Perceived it is harmful	29.6	70.4		29.3	70.7	
**Knowledge that malaria is risky in pregnancy**						
No	27.9	72.1	0.432	12.3	87.7	0.015
Yes	29.5	70.5		27.0	73.0	
**Knowledge that ITN prevents malaria**						
No	21.9	78.1	<0.0001	17.4	82.6	<0.0001
Yes	49.6	50.4		36.6	63.4	
**Knowledge of causes of malaria**						
Others	23.6	76.4	0.007	24.5	75.5	0.765
Mosquito bites	30.1	69.9		25.9	74.1	
**Misconception about causes of malaria**						
No misconception	29.5	70.5	0.408	26.5	73.5	0.565
Had misconception	27.9	72.1		24.6	75.4	
**Knowledge of prevention of malaria**						
Not correct knowledge	23.9	76.1	0.001	19.4	80.6	0.030
Correct knowledge	31.0	69.0		27.8	72.2	
**Misconception about prevention of malaria**						
No misconception	29.7	70.3	0.410	23.5	76.5	0.220
Had misconception	28.1	71.9		27.6	72.4	

**Total**	**28.8**	**71.2**		**25.7**	**74.3**	

### Bivariate analysis of use of ITNs and independent variables

Of the 677 pregnant women who owned bednets (out of 2,348 sampled), only one-quarter (25.7%) of the net owners used bednets the night before the survey. In this section we explore, at the bivariate level, the differences between net owners who use and those who do not use bednets. In terms of background characteristics, there was a significant difference in net use (*p *< 0.0001) between urban (32.1%) and rural residents (20.7%). Among pregnant women who owned bednets, no significant differences in use of bednets were found regarding age, education and geopolitical zones of respondents.

Knowledge about the cause of malaria (*p *= 0.765), misconception about causes (*p *= 0.565), and misconceptions (an index variable) about prevention were not statistically significantly associated with ITN use but knowledge about prevention (an index variable) (*p *= 0.030) was. Thus respondents who had correct knowledge about how malaria can be prevented were more likely to use ITNs; and specifically those who knew that ITNs can prevent malaria were more likely to use them than those who did not (36.6% vs. 17.4%; *p *< 0.0001). Given the dangers of malaria in pregnancy, specific questions were asked to find out how knowledgeable respondents were about such risks, and more importantly, whether those who knew about the risks were more likely to use ITNs. Our results show that pregnant women who knew about the specific risks of malaria in pregnancy (such as anaemia, low birth weight, abortion) were more likely to use ITNs than those who did not (*p *= 0.015). As a sequel, pregnant women who perceived malaria in pregnancy to be harmful were more likely to use ITNs compared with those who did not perceive it to be harmful 29.3% vs. 20.3%; *p *= 0.009)). It was also found that among pregnant women who own bednets, there was no significant difference in bednet use between women who have registered for antenatal care and those who have not (*p *= 0.143).

### Multivariate analysis

Separate analyses were undertaken for each of the two outcome variables: 'net ownership' among all pregnant women (N_1 _= 2,348) and 'ITN use' among bednet owners (N_2 _= 677). In an attempt to simultaneously evaluate possible impact on each of the two outcome variables, by personal level variables and controlling for other covariates, multiple logistic regression was employed. The results are presented in Table 3 showing statistical tests of significance for the relationship between the two outcome variables of interest and the explanatory variables considered in this paper.

**Table 3:** Multivariate analysis of determinants of bednet ownership and use among pregnant women

Variables	Analysis of ownership of bednets among all respondents (N_1 _= 2348)				Analysis of use of ITN among only PW who owned a bednets (N_2 _= 677)			
	
	95% CI for Odds Ratio				95% CI for Odds Ratio			
	
	Odds Ratio	Sig	Lower	Upper	Odds Ratio	Sig	Lower	Upper
Urban	0.805*	0.029	0.663	0.978	1.866*	0.001	1.284	2.712
ageto25	1.105	0.306	0.913	1.338	0.703	0.064	0.484	1.021
Qur'anic	1.374	0.063	0.983	1.921	1.059	0.872	0.529	2.118
Primary	1.276	0.110	0.946	1.720	1.568	0.136	0.868	2.832
Secondary	1.155	0.314	0.872	1.531	0.992	0.977	0.562	1.751
Higher	1.309	0.178	0.884	1.936	1.289	0.485	0.632	2.627
Knowledge of causes of malaria	1.090	0.561	0.815	1.459	0.686	0.221	0.375	1.254
Misconception about causes of malaria	1.010	0.930	0.817	1.247	0.806	0.305	0.535	1.216
Knowledge of malaria prevention	0.807	0.113	0.619	1.052	0.945	0.855	0.517	1.727
No Misconception about prevention	0.977	0.826	0.792	1.205	1.551*	0.036	1.029	2.339
Knowledge that ITN prevents against malaria	3.850*	<0.0001	3.073	4.824	2.931*	<0.0001	1.864	4.610
Knowledge that malaria is harmful in pregnancy	1.281	0.166	0.903	1.817	2.006	0.124	0.827	4.864
Knowledge of risks of malaria in pregnancy	0.832	0.092	0.671	1.031	1.211	0.369	0.798	1.837
Registered for ANC	1.340*	0.003	1.105	1.626	1.192	0.364	0.816	1.740

*Statistically significant difference at *p < 0.05*

A key objective of this study is to identify key mutable variables (for programmatic purposes) with strong explanatory powers on ownership and use of ITNs, even after controlling for other factors. Three predictors of ITN ownership use were identified: residence, knowledge that ITNs prevents against malaria, and registration for antenatal care. Even after controlling for key demographic and other knowledge and risk indicators, pregnant women who live in urban areas were 0.81 times less likely to own bednets compared with pregnant women in rural areas (OR = 0.805, 95% confidence interval 0.663 to 0.978; *p *= 0.029). Knowledge about the efficacy of ITNs in preventing malaria was found to be a strong predictor to bednet ownership. Pregnant women who knew that ITNs prevent against malaria were nearly four times more likely to use ITNs than those who did not have such knowledge (OR = 3.9, 95% confidence interval 3.073 to 4.824; *p *< .0001). Also pregnant women who had registered at antenatal care clinics were 1.3 times more likely to own bednets compared with those who have not (OR = 1.34, 95% confidence interval 1.105 to 1.626; *p *= 0.003).

Since women who do not own bednets are highly unlikely to use them, we limited the analysis on 'use of ITNs' to only pregnant women who own bednets (N = 677); and the results are again shown in Table 3. Pregnant women who live in urban areas were nearly twice more likely to use bednets (OR = 1.866, 95% confidence interval 1.284 to 2.712; *p *= 0.001). Also pregnant women who did not hold misconceptions about prevention of malaria were one and a half times more likely to use ITNs than those who did (OR = 1.551, *p *= 0.036. The explanatory indicator with the highest predictive power on bednet use among pregnant women who owned bednets is knowledge that ITNs prevent against malaria. Women with this knowledge were nearly three times more likely to use bednets compared with those who did not (OR = 2.931, 95% confidence interval 1.864 to 4.610; *p *< 0001);

## Discussion

This survey showed a low rate of ITN ownership, with only 29% of pregnant women reporting that they owned bednets. After controlling for other variables, whether or not a pregnant woman owns a bed depended greatly on three explanatory variables. Women who register at antenatal clinics are more likely to own bednets. This may be the result of free or subsidised distribution of bednets to pregnant women at such facilities. The finding that women in rural areas are more likely to own bednets may reflect the successful penetration of the massive community level distribution campaigns in rural areas. This is supported by the finding that the proportions of pregnant women who own bednets are higher in predominantly rural geopolitical zones including North West and North East than the highly urbanised South West. But by far the explanatory variable with the best predictive power on ITN ownership is knowledge that ITNs prevent against malaria. Pregnant women who knew this were nearly four times as likely to own bednets compared with those who did not. It is important to note that the educational level of pregnant women was not related to ITN ownership.

It is important to note that only 25% of pregnant women who owned ITNs actually slept under one the night before the survey. While this proportion is far higher than the 7.5% for the entire sample, it shows that ownership of ITN does not necessarily translate to use. The low rate of use of ITNs by pregnant women (7.5%) is far below global and national coverage targets. This rate is a marginal improvement over results of earlier surveys with ITN utilizations rates among pregnant women of 1.3% and 2.9% in 2003 and 2006 respectively [10,11]. The 2008 Nigerian Demographic and Health Survey also showed ITN utilization rate below 10%, further corroborating recent observations that ITN utilization in Nigeria has remained consistently low [13]. The use of ITN in many malaria endemic of sub-Saharan Africa has remained generally low as shown by a recent synthesis of national data sets from several countries [15].

Our study explored bednet use among pregnant women who owned bednets. This was necessary given that several studies have shown that within households that owned ITNs, several under-five children and pregnant women did not sleep under them [13,16-18,26]. In one report, as much as 55% of children in households that owned ITN did not sleep under ITN [16]. Within-country and between-country analysis of data on net ownership and use from selected sub-Saharan African countries show wide gaps between ITN ownership and use.

Our study has identified several determinants of the use of ITNs by pregnant women. Several factors influencing ITN utilization in pregnancy have also been identified by other Nigerian authors. These include education, place of residence (locality) and access to antenatal care services [27-29]. Other studies have shown that educational level positively influences care-seeking behaviour. A study of pregnant Ethiopian women showed that higher educational attainment and residence in urban location were significant predictors of ITN use in pregnancy [30]. Our study however showed no significant influence of educational attainment on ITN at both bivariate and multivariate levels suggesting the likely dominance of yet unclear, socio-cultural covariates of education in our setting. Residence in urban areas was, however, confirmed as a predictor of ITN use in our study.

Our study identified two key knowledge-based predictors of ITN use in pregnancy among women who own bednets. Pregnant women who knew that ITNs prevent against malaria were three times more likely to use bednets compared with those who did not. Similarly women who held no misconceptions about malaria prevention were more likely to use ITNs.

When the findings on ITN ownership are juxtaposed with net use, there are some key findings worth discussing. While it was found that pregnant women in rural areas were more likely to own ITNs (apparently as a result of mass community level distribution), in terms of use, pregnant women in urban areas are nearly twice as likely to use. This seems to suggest that a higher proportion of women in rural areas who own bednets do not use them. When one considers that a higher proportion of pregnant women in Nigeria live in rural areas, the enormity of the task becomes clearer to malaria prevention health promotion professionals. Another key finding is that while registration at antenatal clinic is a key predictor for ITN ownership, when it comes to ITN use, there is no difference between pregnant women who have registered at antenatal clinics (and presumably have been given free bednets) compared with those who have not registered. The only indicator that remained strong in explaining both ITN ownership and ITN use is the knowledge that ITNs prevent against malaria. Pregnant women who knew this were nearly four times more likely to own nets and nearly three times more likely to sleep under one. In terms of programming, therefore, efforts are needed to expand community level and antenatal distribution campaigns to intensify appropriate behaviour change interventions that emphasize the efficacy of ITNs in malaria prevention.

In our study ITN use is lagging behind ownership by quite a wide margin. With the recent massive distribution of ITNs in Nigeria, access is expected to invariably go up and may attain the national target but the extent to which this would improve utilization is being cautiously watched. As has been shown in this study, it is widely acknowledged that increase in ITN access (i.e. household ownership) does not necessarily translate to commensurate increase in utilization. It has been hypothesized that the gap between ITN access and utilization would be reduced significantly only when access at the household levels reaches or exceeds one ITN for two persons [26]. This underscores the need to increase the number of ITNs available to users in a given household. This seems to suggest that the current mass distribution of ITNs may not necessarily lead to use unless it is accompanied with behaviour change interventions that address the community level perceptions, misconceptions and positively position ITN as an effective prevention device to prevent malaria.

The link between personal risk perception and risk-reduction behaviour has been well researched in health promotion [31,32]. The perception of risk may play a significant role in the decisions of individuals to use ITNs [33,34]. At the bivariate level, it was found that pregnant women who knew about the specific risks of malaria in pregnancy (such as anaemia, low birth weight, abortion) were more likely to use ITNs than those who did not. Similarly pregnant women who perceived malaria in pregnancy to be harmful were more likely to use ITNs compared with those who did not perceive it to be harmful. Pregnant women who do not see themselves or their unborn babies at risk for malaria, are less likely to use preventive devices such as ITNs, even if they possess them. For both pregnant women who own ITNs and those who do not, the knowledge that malaria may be harmful to the outcome of the pregnancy is a significant predictor of ITN use. Health promotion programmes should consider including messages that ask pregnant women to consider actual risk reduction accruable from using ITNs to protect her unborn child even if there are potential inconveniences for the woman herself.

It is important to note some limitations of this paper. Given that ITNs were distributed free in the study states, the study did not obtain information on how ITNs were acquired or the cost, factors which may affect ITN ownership in different circumstances. Also the study did not explore the physical attributes of ITNs in explaining user preferences and the potential influence on consistent ITN use. Since women will use bednets only when they owned one, we limited our study to the 21, out of 36 states, where there are current efforts at mass distribution of bednets; hence the findings may not necessarily apply to the whole country.

## Conclusions

Ownership of bednets among pregnant women was found to be very low- only 28.8%. Three key variables explained differences in ITN ownership. Women who live in rural areas were more likely to own nets as well as those who had registered at antenatal clinics. Both explanatory variables may be the result of high profile community level ITN distribution campaigns. The third variable, also amenable to programmatic intervention, is the knowledge that ITNs prevent against malaria. Efforts to achieve universal household ownership of bednets for pregnant women may need to consider addressing, among others, these three explanatory variables. The use of ITNs by pregnant women in Nigeria was very low even when compared with other African countries. Only 7.5% of pregnant women slept under an ITN the night before the study. Also there was a wide gap between ITN ownership and use. Only 25.7% of pregnant women who owned ITN used them. Among pregnant women who owned bednets, those who knew that ITNs prevent against malaria were more likely to use them. While rural women were more likely to own nets, it was found that they were less likely to use them compared with urban women. Also registration at antenatal clinics ensured that pregnant women owned ITNs (probably as a result of free or subsidized distribution of ITNs at these facilities) but in terms of its use, there is no difference between pregnant women who register at antenatal clinics and those who do not. It is apparent accessibility alone is not sufficient, and that distribution campaigns at both community and clinic or health facility level may not necessarily translate ITN ownership to ITN use.

Pregnant women who knew that malaria could be prevented with ITNs were more likely to use ITNs. Also those who knew that malaria is harmful in pregnancy were more likely to use ITN. The link between personal risk appraisal and risk-reduction behaviour has been well researched in health promotion. The perception of risk may play a significant role in the decisions of individuals to use ITNs. If pregnant women did not see themselves or their unborn babies at risk for malaria, they are less likely to use preventive devices. In terms of programming, therefore, in addition to expanding household bed net ownership, efforts should be placed on behaviour change messages that aim at improving knowledge about the efficacy of ITNs in malaria prevention and addressing misconceptions related to prevention. The fact that neither ITN ownership nor use is related to level of education demands that all pregnant women both literate and well-educated should be targets of ITN ownership and use. It is important that those who plan behaviour change interventions or malaria control in pregnancy may consider these factors in the design and implementation of behaviour change communication strategies.

## Competing interests

The authors declare that they have no competing interests.

## Authors' contributions

All authors were involved in conduct and reporting of the survey. Development of the study design and implementation was conducted by JA, EDA, EN, OL, SBA and AA. Data were analyzed by SBA, AA, EDA and MMM. All authors contributed to drafting and completion of the manuscript. All authors read and approved the final version of the manuscript.

## Pre-publication history

The pre-publication history for this paper can be accessed here:

http://www.biomedcentral.com/1471-2458/12/105/prepub
